# VOLUNTEERISM AND PURPOSE IN LIFE IN A NATIONAL SURVEY OF COMMUNITY-DWELLING MIDDLE-AGED AND OLDER ADULTS

**DOI:** 10.1093/geroni/igad104.3009

**Published:** 2023-12-21

**Authors:** Wingyun Mak, Orah Burack, Margret Howard, Kenneth Boockvar

**Affiliations:** The New Jewish Home Research Institute on Aging, New York City, New York, United States; The New Jewish Home Research Institute on Aging, New York City, New York, United States; The New Jewish Home Research Institute on Aging, New York City, New York, United States; University of Alabama Birmingham, Birmingham, Alabama, United States

## Abstract

Volunteerism has been shown to provide physical, psychological, and functional health benefits as people age. Volunteers are able to participate in meaningful service, develop social connections, and exercise their abilities. In this study, we examined whether engaging in various types of volunteer engagement (e.g. charitable, political, religious, environmental, animal welfare, support group) was associated with self-perceived change in purpose in life (PIL) over a five year lookback period, controlling for demographic and health variables. Data were drawn from the AARP Social Engagement and Brain Health Survey, which included a nationally representative sample of adults aged 40-75+ (mode=55-64 years) across the United States who completed an online survey (N=2,585). Hierarchical linear regression was used. People who were younger (β=-.06, t=-2.55, p=.01), Black (β=.23, t=10.50, p<.001) or Hispanic (β=.12, t=5.20, p<.001), married (β=.05, t=2.65, p=.01), and had better health (β=.16, t=7.09, p<.001) and cognition (β=.18, t=7.69, p<.001) reported greater increase in PIL over the last five years (ΔR2=.13, p<.001). After controlling for demographic and health variables, people who valued PIL (β=.15, t=7.49, p<.001; ΔR2=.02, p<.001) and participated in volunteer work through a religious organization (β=.05, t=2.38, p=.02) or support groups (β=.07, t=3.10, p=.002) were more likely to report greater levels of PIL over the last five years (ΔR2=.02, p<.001). These results suggest that changes in experienced PIL may depend on physical and cognitive health, importance placed on PIL, and type of volunteer engagement, all of which may be points of potential intervention.

